# Supplementary figures and images for: Deletion of a putative promoter-proximal Tnfsf11 regulatory region in mice does not alter bone mass or Tnfsf11 expression in vivo
Source: PLoS One. 2021 May 10;16(5):e0250974. doi: 10.1371/journal.pone.0250974 (PMC8109787; doi:10.1371/journal.pone.0250974)

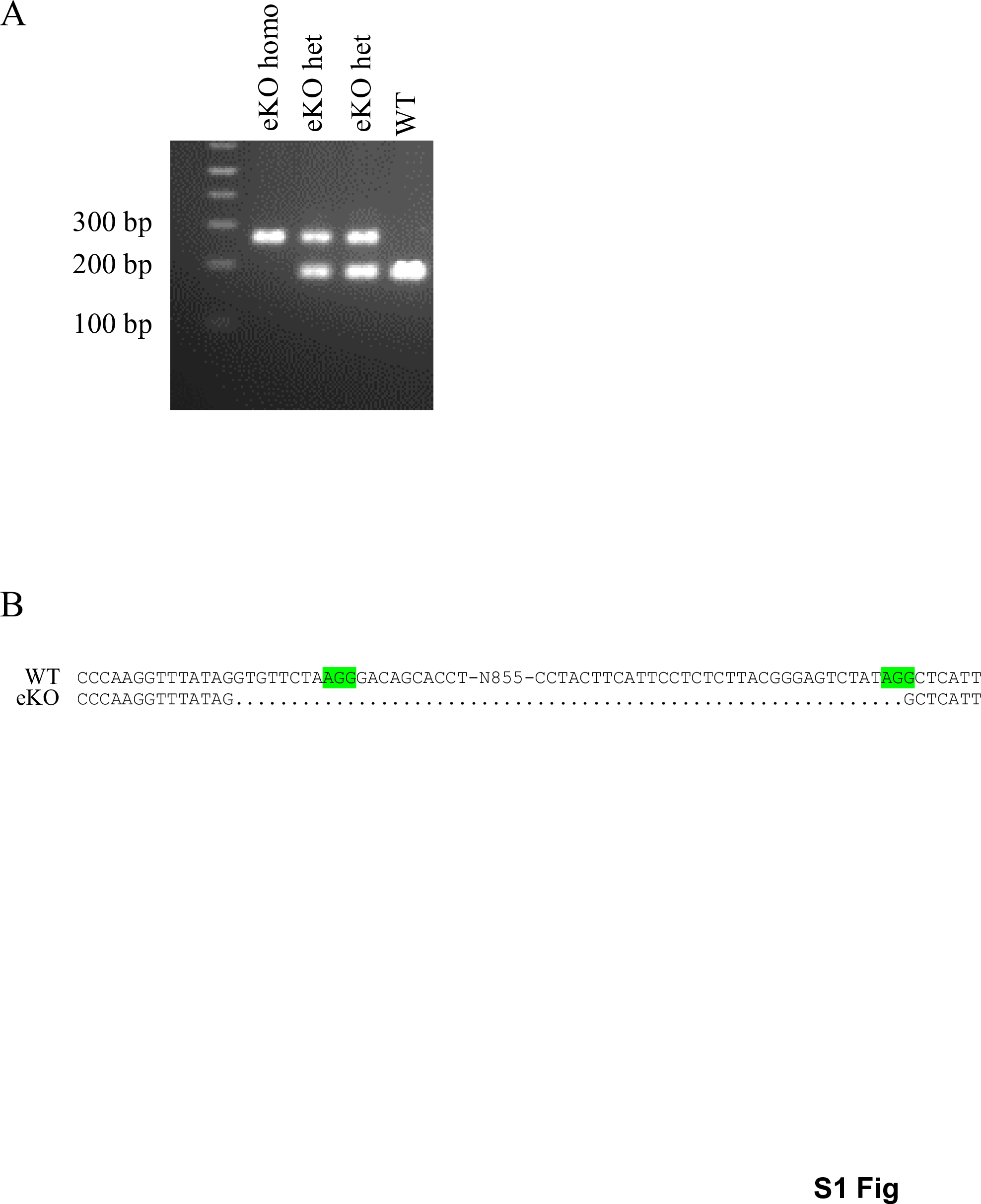

Supplement: S1 Fig — (A) PCR products from the genotyping PCR described in the Material and methods section were fractionated on an agarose gel, stained with ethidium bromide, and imaged. The size of the eKO product is 261 bp and that of the WT product is 185 bp. Representative results are shown for a homozygous eKO mouse, heterozygous eKO mice, and a WT mouse. (B) The sequence of the eKO allele was determined by TA-cloning of the 261 bp PCR product and sequencing. The relevant portion of the sequencing result is shown aligned to the sequence of the WT allele. 855 bp of the WT allele were ommitted from the figure to reduce the size of the sequence. The PAMs for each of the sgRNAs used to create the deletion are highlighted in green. (TIF) [file pone.0250974.s001.tif]

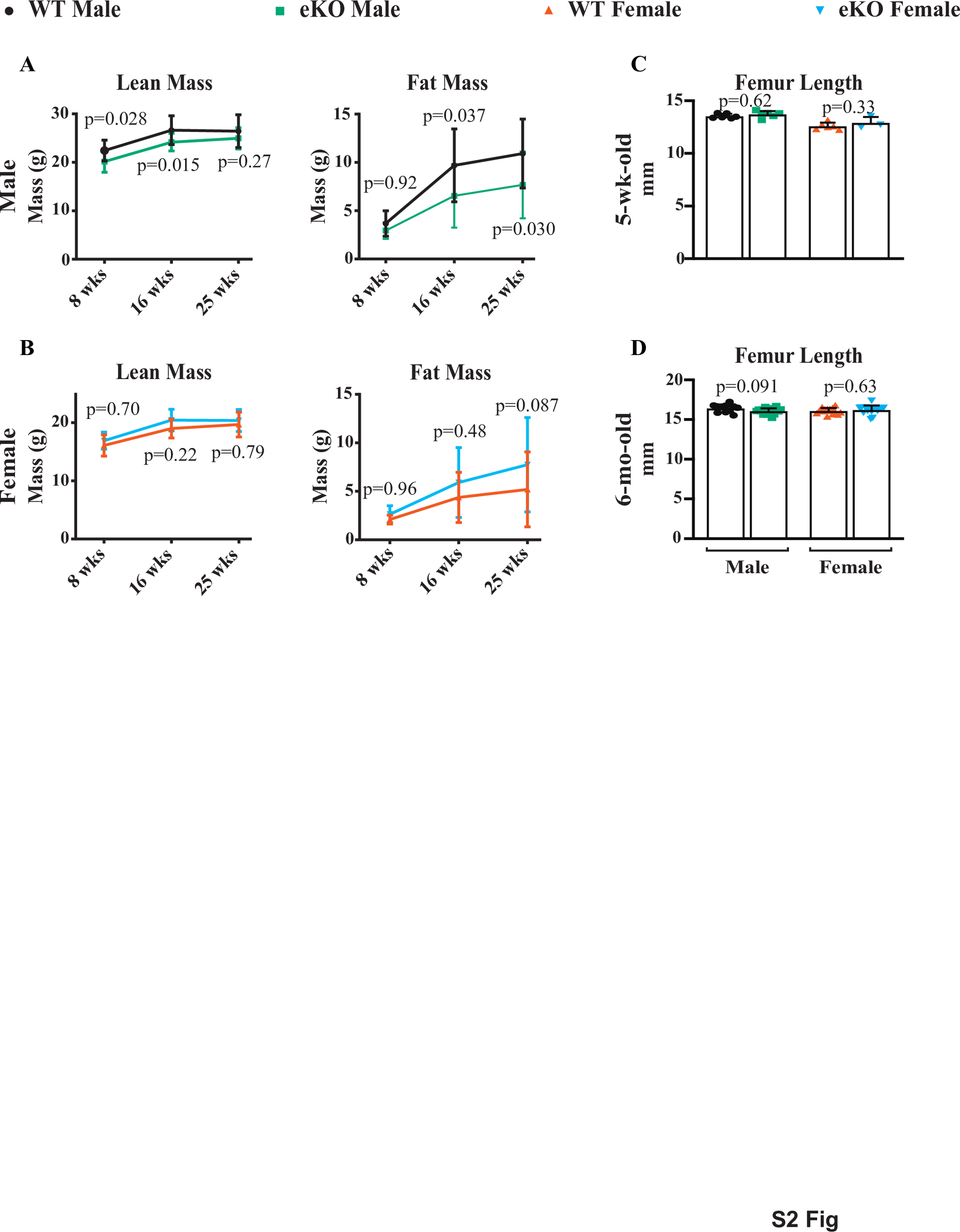

Supplement: S2 Fig — Serial analysis of lean and fat mass using Piximus-derived fat percentage and total tissue mass data beginning at 8 weeks of age until 25 weeks of age in male (A) and female (B) wild type and homozygous eKO mice. n = 11 to 18 per group, p values determined using 2-way ANOVA. (C-D) Femur length of 5-week-old and 6-month-old mice using right femurs measured with calipers. n = 3 to 18 per group, p values determined using 2-way ANOVA. Values are means ± s.d.. (TIF) [file pone.0250974.s002.tif]

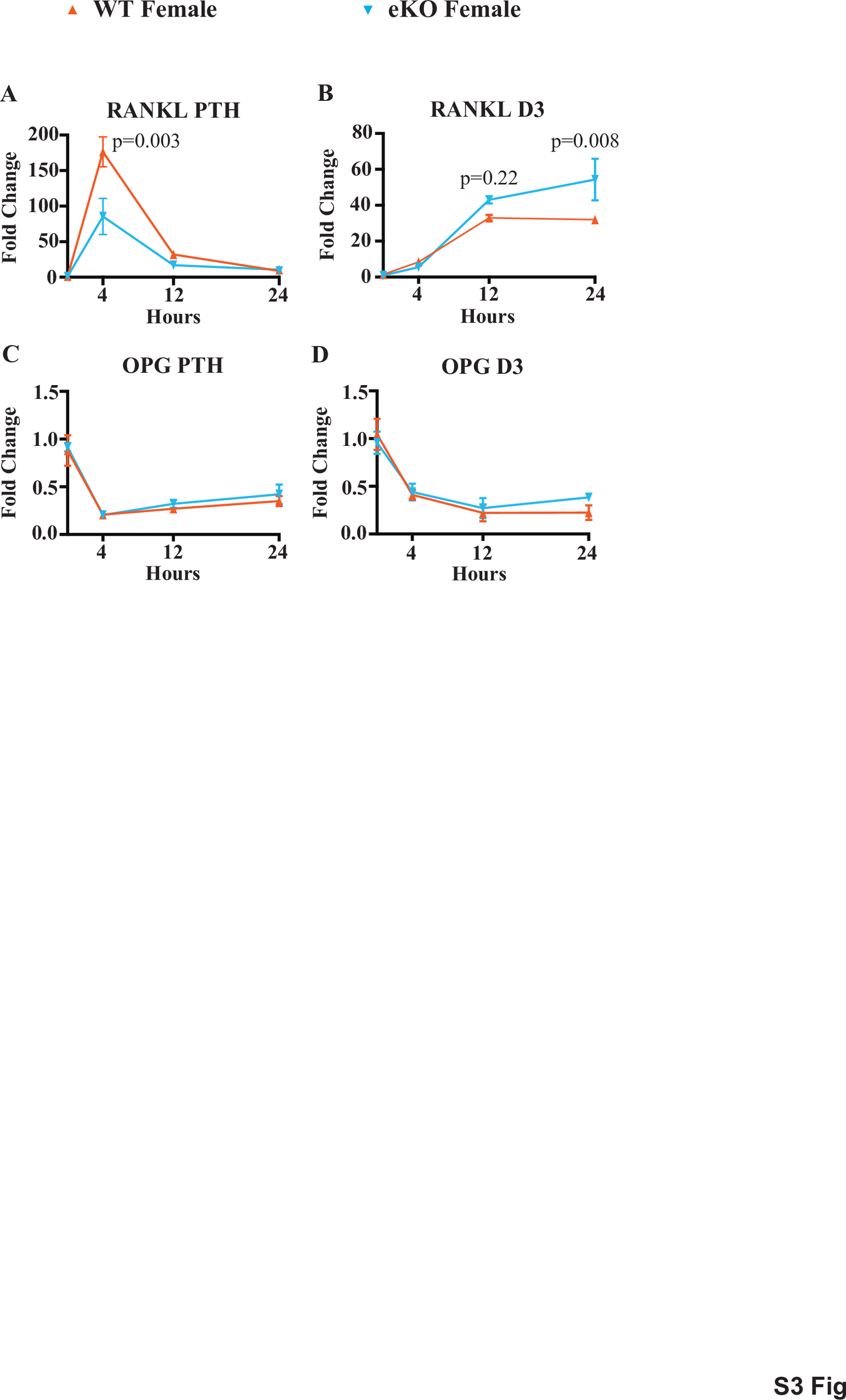

Supplement: S3 Fig — (A-D) Tnfsf11 (RANKL) and Tnfrsf11b (OPG) mRNA measured by Taqman RT-PCR in RNA prepared from bone marrow stromal cell cultures from female mice of the indicated genotype treated with vehicle, 10−7 M PTH, or 10-8M 1,25(OH)2D3 for the indicated times. Values are the mean fold-change, ± s.d., of triplicate wells; p values determined by comparing eKO versus WT values at the same time point by Repeated Measures Mixed Effects models. (TIF) [file pone.0250974.s003.tif]

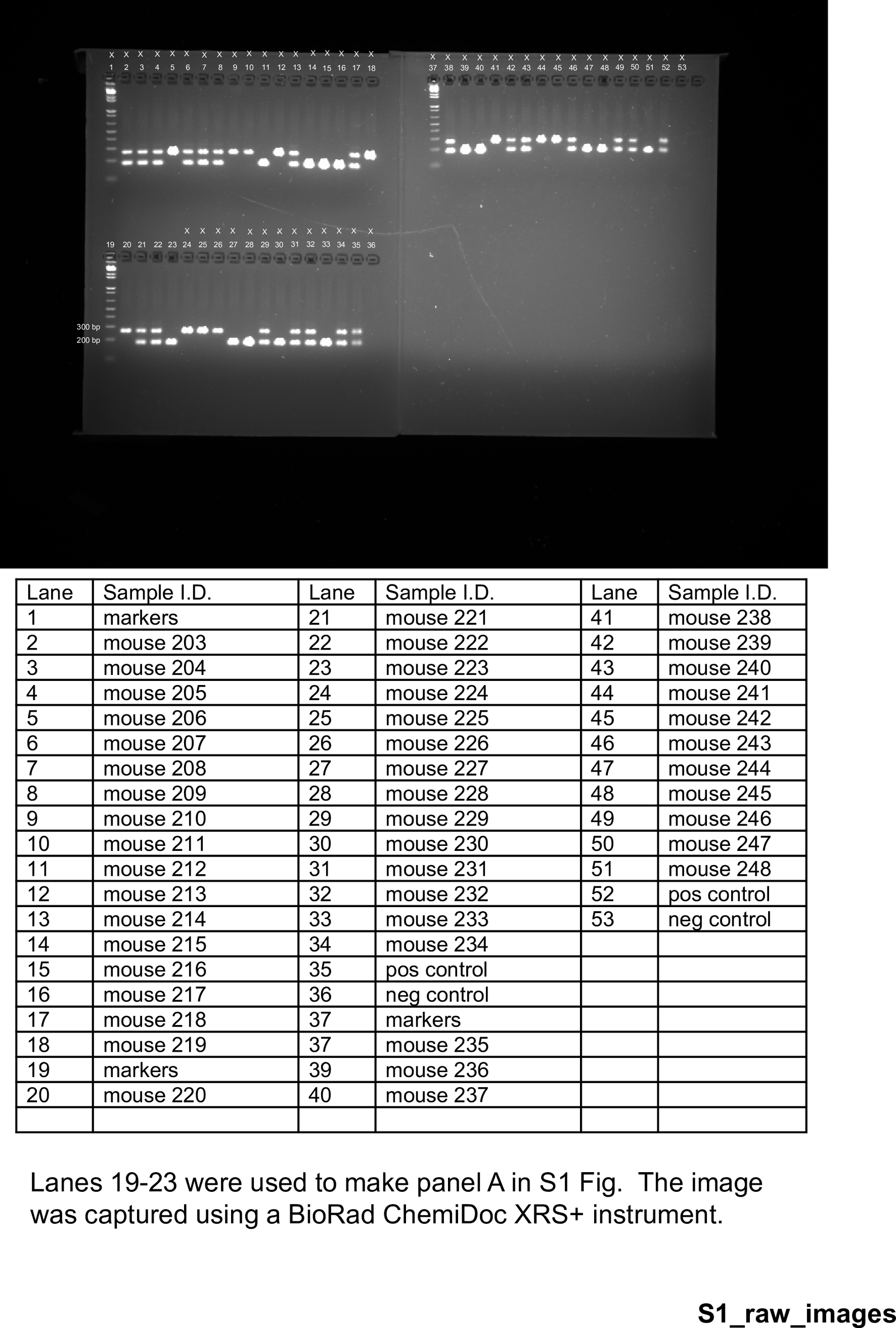

Supplement: S1 Raw images — (TIF) [file pone.0250974.s004.tif]
